# Acute perimyocarditis mimicking transmural myocardial infarction

**DOI:** 10.1186/1755-7682-2-37

**Published:** 2009-12-09

**Authors:** Hesham R Omar, Ahmed Fathy, Rania Rashad, Mohamed Elghonemy

**Affiliations:** 1 Department of Cardiology, Cairo University Hospital, Cairo, Egypt; 2 Department of Cardiology, National Heart Institute, Cairo Egypt; 3 Department of Critical Care Medicine, Cairo University Hospital, Cairo, Egypt

## Abstract

Although acute pericarditis has charachteristic electrocardiographic (ECG) findings that differentiate it from acute ST segment elevation myocardial infarction (MI); in certain cases diagnosis is somewhat difficult especially when the ECG reveals focal instead of diffuse changes and moreover when pericarditis is associated with an underlying myocarditis causing elevation of the cardiac biomarkers therefore increasing the difficulty in differentiating between both enteties. This is especially important because adverse lethal side effect can occur if thrombolytic therapy is administered for a patient with acute pericarditis, or if a diagnosis of transmural MI is missed. In this case report we are describing an 18 year old male patient who presented with an acute onset of severe chest pain associated with focal ECG changes and elevated cardiac enzymes mimicking transmural MI. This report aims to sensitize readers to this debate and create awareness among cardiologists and intensivists with both presentations and how to reach an accurate diagnosis.

## Introduction

It's not uncommon to find the coexistence of both pericarditis and myocarditis since both are commonly caused by cardiotropic viruses. There is a wide variety in clinical presentation reflecting the extent of myocardial involvement. Some cases might pass asymptomatic probably masked by symptoms of the preceeding illness, and some cases may be severe leading to heart failure necessitating inotropic support or even transplantation. Due to the underlying myocardial involvement some patients may present with ST segment elevation on the resting ECG and elevated cardiac biomarkers mimicking acute transmural myocardial infarction. Therefore, perimyocarditis would be difficult to diagnose without detailed history taking and proper electrocardiographic interpretation. In this case report we will present an 18 year old patient with perimyocarditis in whom focal ECG changes and elevated cardiac enzymes simulated acute MI.

## Case Report

An 18 year old male patient presented to the emergency department with chest pain. The patient gave an 8 hours history of intermittent retrosternal chest pain which occurred at rest and was referred to the left shoulder with a severity of 8/10. The pain had an acute onset, progressive course and a compressing nature. The patient presented to our department after he sought medical advice at an out-patient clinic where he received non steroidal anti-inflammatory drugs without relief of pain. There was no history of smoking, diabetes, hypertension, dyslipidemia, a positive family history of coronary artery disease or history of cocaine abuse. The patient reported no symptoms suggestive of a preceding viral illness. Clinical examination revealed B.P. 110/80 mmHg, pulse 90/min, temperature 37.6°C. Cardiac examination revealed normal S1, S2, with no additional sounds, murmurs or pericardial rub. The rest of physical examination was normal.

**Figure 1:**
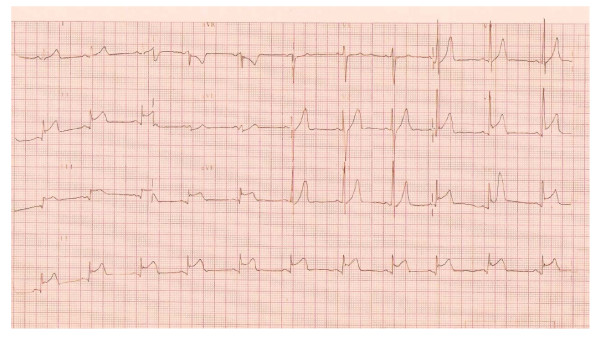
**Showing concave upwards ST segment elevation in leads II, III, Avf, V5 and V6**. There is no evidence of PR segment depression.

The ECG performed in the emergency department was abnormal and a preliminary diagnosis of inferolateral myocardial infarction was presumed. The patient was then admitted to the coronary care unit. ECG revealed ST segment elevation in leads II, III, aVF, and V5-6 with no evidence of PR segment depression as shown in figure 1.

Laboratory investigations revealed an elevated TLC of 13000, ESR first hour 45, The first set of cardiac enzymes was elevated CK 418, CKMB 56 and Troponin I 3.5. The laboratory investigations were otherwise normal.

The diagnosis was a matter of debate where two conflicting opinions ensued. The first opinion suggested acute perimyocarditis based upon the patient's age, absence of risk factors of coronary artery disease, low grade fever on presentation and the coved upward pattern of the ST segment and consequently suggested NSAID as the mainstay of treatment. The second opinion suggested ST segment elevation MI based upon the focal ECG findings and elevated cardiac enzymes consequently favoring the use of thrombolytic therapy for treatment.

Taking into consideration the urgency of making the distinction to avoid the harms of missing the diagnosis, we decided to perform an urgent bedside echocardiography. Transthoracic echocardiography was normal with no evidence of regional wall motion abnormalities, normal biventricular systolic function and no evidence of pericardial effusion. This favored the diagnosis of acute perimyocarditis.

Indomethacin 50 mg t.d.s was started with marked improvement noticed on the second day of treatment. The patient experienced a smooth hospital course with fever reaching 38.5 and occasional chest pains as his main complaints. On the fourth day of hospital admission the patient became completely symptom free and was discharged from the CCU on indomethacin 50 mg t.d.s with a recommendation of restricting his physical activity for 4 weeks.

Two days after discharge the patient re-experienced the same chest pain and sought medical advice at the National Heart Institute where he was adviced to perform a diagnostic coronary angiography to rule out the presence of an underlying coronary artery disease. The patient refused to perform coronary angiography so exercise ECG was performed and was negative for exercise induced myocardial ischemia.

## Discussion

Perimyocarditis is an acute inflammation of the pericardium and the underlying myocardium resulting in myocellular damage. It is usually asymptomatic with complete resolution in most cases. It can however lead to fulminant cardiac failure resulting in death or requiring cardiac transplantation.

Establishing the underlying aetiological pathogen is of utmost importance as it may alter the treatment and outcome. Perimyocarditis is most commonly of viral aetiology predominately the coxsackie B virus. However, other viruses have been incriminated as the cause. These include cytomegalovirus [1] parvovirus B 19 [2] epestein barr virus [3] Rubella [4] influenza A virus [5] and during hepatitis A virus infection [6].

A large group of bacterial pathogens have also been described as possible etiological agents. The most reported bacterial pathogens are borrelia burgderferi [7] and campylobacter jejuni [8]. Other bacterial pathogens include mycoplasma pneumonia [9], Chlamydia pneumonia [10], brucella [11], Rickettsia Helvetica [12], yersinia enterocolitica [13], ricketsial q fever [14], shigella boydii [15], sonnei [16] and tuberculosis [17]following streptococcal tonsiliitis [18]and meningococcal septicemia[19]The protozoan toxoplasma gondii [20] has also been reported as a cause.

Certain immunizations have been linked with perimyocarditis. The vaccination that has received great attention recently is the smallpox vaccine, particularly after its reinstitution for military personnel in 2002 and the report of 50 cases of probable myocarditis temporally related to it [21,22] There is 1 reported case of myocarditis that developed hours after diphtheriatetanus-acellular pertussis (DTaP) vaccination in a 3-month-old [23] and another case of myocarditis after tetanus vaccination alone in a 14-year-old [24]. perimyocarditis has also been linked to the administration of certain drugs including meselazine therapy used in the treatment of inflammatory bowel disease [25].

The clinical presentation is varied, reflecting the variability of myocardial involvement that may be focal or diffuse, affecting any or all cardiac chambers. Many cases may be subclinical where subtle cardiac symptoms and signs may be overshadowed by the systemic manifestations of the viral infection. While other cases manifest by the full blown picture of heart failure.

Some patients with perimyocarditis can present with acute chest pain associated with ST segment elevation on ECG and elevated cardiac enzymes, therefore mimicking acute transmural myocardial infarction. A thorough and a detailed history, examination and ECG interpretation by an expert cardiologist is mandatory in this situation to avoid any complications that may arise in cases of inaccurate diagnosis.

A detailed history should take into cinsideration the patients age, underlying medical problems including diabetes, hypertension, dyslipedemia, smoking, positive family history of coronary artery disease and cocaine abuse that can place the patient at risk for myocardial infarction. History should also include character of pain, relation to cough, inspiration, posture or deglutition and radiation which is classically to the trapezius ridge in cases of pericarditis. A previous viral illness as well as fever should be searched for which if present will favour the diagnosis of pericarditis.

Electrocardiogram is of utmost importance in differentiation between the two enteties. ST segment elevation in pericarditis is classically diffuse involving all leads except aVR and V1, and is charachteristically concave upwards without reciprocal changes differentiating it from ischemia. However ST segment elevation in pericarditis is sometimes focal in which the distinction is most difficult. PR segment depression is another distinguishing feature which sometimes occurs in the absence of ST segment elevation and can be the intial clue for clinching the diagnosis [26]. Later on, days to week after diagnosis of perimyocarditis, T waves are inverted only after normalization of the ST segment to the isoelectric point which is not the case in ST elevation MI where T waves can invert while the ST segment is still elevated.

Several reports have been previously published demonstrating the similarities between both diagnoses in their presentations [27,28]. In our case, there were some clues that favoured perimyocarditis including the young age of the patient, absence of any risk factors for coronary artery disease, low grade fever on admission, concave upwards ST segment elevation and the absence of regional wall motion abnormalities on transthoracic echocardiogram. However the elevated cardiac enzymes and the focality of ECG findings were somewhat distracting making the diagnosis of perimyocarditis notoriously difficult necessitating an expert cardiologist to ensure an accurate diagnosis.

Acute perimyocarditis can also be differentiated from acute MI by contrast-enhanced cardiovascular MRI. In acute myocarditis, myocardial late gadolinium enhancement is present in up to 88% of cases [29,30] which characteristically has patchy distribution not conforming to any particular coronary territory and usually in the subepicardial and not the subendocardial layer [31] thus differentiating it from MI. Another option to exclude coronary artery disease noninvasively is the use of the widely available 64-slice CT coronary angiography which has shown a very high negative predictive value for excluding coronary artery disease.

In conclusion, we are reporting a case of perimyocarditis presenting with focal ST segment elevation and elevated cardiac enzymes mimicking transmural myocardial infarction. A thorough and detailed history taking and proper ECG interpretation is mandatory to reach an accurate diagnosis and avoid any complication that may arise if the correct diagnosis is missed. Due to the undesired complications of acute myocarditis including serious ventricular arrhythmias, ventricular dilatation and heart failure which can be life threatening, we assume that the diagnosis of myocarditis is more important than pericarditis. Monitoring of the cardiac biomarkers is therefore of utmost importance in any patients presenting with classic symptoms and ECG findings of acute pericarditis to exclude an underlying myocarditis. After hospital discharge, patient should be followed for several weeks to exclude the development of heart failure or subclinical left ventricular dysfunction.

## Abbreviations

ECG: electrocardiography; MI: myocardial infarction; CCU: coronary care unit; NSAID: nonsteroidal anti-inflamatory drugs; MRI: magnetic resonance imaging.

## Consent

Written informed consent was obtained from the patient for publication of this case report. A copy of the written consent is available for review by the Editor-in-Chief of this journal.

## Competing interests

The authors declare that they have no competing interests.

## Authors' contributions

HO was responsible for diagnosis of the case. HO was responsible for literature search. HO and AF were responsible for drafting the manuscript. RO and ME have made critical revisions to the manuscript. All authors have read and approved the final Manuscript.
